# Real-time PCR assays for genotyping of *Cryptococcus gattii* in North America

**DOI:** 10.1186/1471-2180-14-125

**Published:** 2014-05-13

**Authors:** Erin J Kelley, Elizabeth M Driebe, Kizee Etienne, Mary E Brandt, James M Schupp, John D Gillece, Jesse S Trujillo, Shawn R Lockhart, Eszter Deak, Paul S Keim, David M Engelthaler

**Affiliations:** 1 The Translational Genomics Research Institute, 3051 W. Shamrell Blvd. Ste. 106, Flagstaff, AZ 86001, USA; 2 Centers for Disease Control and Prevention, Atlanta, GA, USA; 3 Department of Pathology and Laboratory Medicine, David Geffen School of Medicine at UCLA, Los Angeles, CA, USA; 4 Center for Microbial Genetics and Genomics, Northern Arizona University, Flagstaff, AZ, USA

**Keywords:** Cryptococcus gattii, Genotyping, Real-time PCR, Epidemiology

## Abstract

**Background:**

*Cryptococcus gattii* has been the cause of an ongoing outbreak starting in 1999 on Vancouver Island, British Columbia and spreading to mainland Canada and the US Pacific Northwest. In the course of the outbreak, *C. gattii* has been identified outside of its previously documented climate, habitat, and host disease. Genotyping of *C. gattii* is essential to understand the ecological and geographical expansion of this emerging pathogen.

**Methods:**

We developed and validated a mismatch amplification mutation assay (MAMA) real-time PCR panel for genotyping *C. gattii* molecular types VGI-VGIV and VGII subtypes a,b,c. Subtype assays were designed based on whole-genome sequence of 20 *C. gattii* strains. Publically available multilocus sequence typing (MLST) data from a study of 202 strains was used for the molecular type (VGI-VGIV) assay design. All assays were validated across DNA from 112 strains of diverse international origin and sample types, including animal, environmental and human.

**Results:**

Validation revealed each assay on the panel is 100% sensitive, specific and concordant with MLST. The assay panel can detect down to 0.5 picograms of template DNA.

**Conclusions:**

The (MAMA) real-time PCR panel for *C. gattii* accurately typed a collection of 112 diverse strains and demonstrated high sensitivity. This is a time and cost efficient method of genotyping *C. gattii* best suited for application in large-scale epidemiological studies.

## Background

Cryptococcosis, a potentially fatal fungal disease, has primarily been observed in immune-compromised individuals and mainly associated with *Cryptococcus neoformans* infection. It is now recognized that *Cryptococcus gattii*, once considered to be a variety of the *Cryptococcus neoformans* complex, is also capable of causing serious disease in immunocompetent individuals and animals [1,2]. *C. gattii* has been associated with a number of tree species in tropical and subtropical regions [3]. More recently, *C. gattii* caused an outbreak that began in 1999 on Vancouver Island, British Columbia and has spread to mainland Canada and the US Pacific Northwest [4]. This outbreak is unique in that it marked the identification of a *Cryptococcus* specie*s* in a new climatic region (from tropical to temperate), habitat (from tropical trees to temperate; e.g., Douglas Fir) and host disease (from primary neurologic to primary pulmonary) [3,5].

Recent epidemiological studies of *C. gattii* in North America provide insight into the organism’s geographical expansion as well as the distribution of molecular genotypes [6-9]. *C. gattii* has been classically classified into four molecular types by MLST/AFLP, VGI/AFLP4, VGII/AFLP6, VGIII/AFLP5, VGIV/AFLP7 [3,5], with additional molecular types recently identified [10]. Interestingly, molecular types have been associated with significant differences in disease type [3,5], antifungal susceptibilities [3,5,10], and severity and outcome [3,5].

Contemporary methods for genotyping *C. gattii* are PCR-restriction fragment length polymorphism (PCR-RFLP), amplified fragment length polymorphism (AFLP), multilocus microsatellite typing (MLMT), multilocus sequence typing (MLST), and most recent, matrix-assisted laser desorption ionization-time-of-flight mass spectrometry (MALDI-TOF MS) [11-14]. High resolution melting (HRM) is a method that has been used to identify the *Cryptococcus neoformans-Cryptococcus gattii* complex, though it has not been employed for genotyping within either species [15]. PCR-RFLP and AFLP require extensive lab work involving restriction enzyme digestion and gel electrophoresis [11]. Results are based on interpretation of gel electrophoresis profiles and as such, are not readily transferred or analyzed between laboratories. MLST, which requires DNA sequencing of seven housekeeping genes, is the preferred genotyping method for *C. gattii* and is easily transferrable between laboratories [16]. MLMT allows for finer genotype resolution than MLST and has high reproducibility between laboratories [14]. In some laboratories, real-time PCR is a preferable option to methods involving DNA sequencing (MLMT and MLST), which require either out-sourcing to a sequencing capable laboratory or investment in, and the maintenance of, an in-house instrument. Although MALDI-TOF MS shows promise as a new genotyping method, instrumentation is expensive and thus prohibitive for many public health laboratories. Conversely, real-time PCR instruments are becoming ubiquitous, easily maintained, and the use of unlabeled primers and no probe makes reagents inexpensive [17]. Therefore, real-time PCR is an accessible and increasing popular technology for widespread molecular epidemiological efforts.

Here, we present a panel of real-time PCR assays, based on mismatch amplification mutation assay (MAMA) methodology, for rapid and sensitive molecular genotyping of *Cryptococcus gattii* molecular types (VGI-VGIV) and the dominant North American VGII subtypes (VGIIa-c) [18,19]. MAMA, a form of allele-specific PCR (ASPCR), employs primers that are designed for SNP genotyping. We use known MLST sequences for the VGI-VGIV molecular type assay design and whole genome sequences of 20 strains to identify SNPs specific to each of the targeted VGII subtypes [9,20].

## Methods

### SYBR MAMA design

MAMA primers have an intentional penultimate mismatch nucleotide at the 3′ end; the ultimate base is always the SNP assay target and is a perfect match for the target SNP [18]. Mismatches decrease the efficiency of primer extension by Taq polymerase, such that if two mismatches are found together under the 3′ end of the primer, the efficiency of the PCR is significantly reduced. However, if a single mismatch at the penultimate base is present, extension occurs from the 3′ matched base, and efficiency of the PCR remains relatively high. Costly fluorogenic oligonucleotide probes are not needed to discriminate SNPs with this method. This discriminatory design results in a cost-efficient, powerful and simple method of SNP genotyping [17,21]. Separate PCR reactions are performed with a MAMA primer specific for only one of the two target SNPs and with one universal primer for amplification from the alternate direction. Comparison of cycle threshold (Ct) values will reveal which reaction is more efficient (has the smaller Ct value). The more efficient reaction corresponds to the SNP that is present in the sample.

### MAMA design for MLST groups VGI, VGII, VGIII, and VGIV

The MLST SYBR MAMA design was informed by MLST data collected for 202 *C. gatii* strains from a worldwide collection [20]. The MLST library included sequences from 77, 75, 26, and 24 isolates of the VGI, VGII, VGIII, VGIV molecular types, respectively. The gene encoding *mannitol-1-phosphate dehydrogenase (MPD1)* was selected as the best candidate for assay design based on its sequence conservation within each of the four molecular types that allowed for design of assay primers with a minimum number of degenerate bases. All 15 of the known *MPD1* allele sequences were aligned with SeqMan Pro v.9.0.4 (DNASTAR, Madison, WI). SNPs specific for each of the molecular types were identified in the sequence alignment. MAMA primers were manually designed in Primer Express 3.0 (Life Technologies, Carlsbad, CA) software with optimal mismatches chosen as suggested by Li et. al. [19] (Table 1).

**Table 1:** MAMA real-time PCR assay sequences and targets for genotyping C. gattii

**Genotype**	**Assay Name**	**Gene (SNP position)**	**Base call match/mismatch**	**Universal Primer sequence 5′ -- > 3′**	**Match MAMA Primer sequence 5′ -- > 3′**	**Mismatch MAMA Primer sequence 5′ -- > 3′**
VGI	VGI-MPD471	*MPD1 (471)*	G/A	AGACTGTCCCAATGTCAAGCTTTC	GCCTTGTATGTGGTAACACCAGTG	GWGCCTTGTATGTGGTAACACCAGTA
VGII	VGII-MPD495	*MPD1 (495)*	T/A	AGACTGTCCCAATGTCAAGCTTTC	ATTAACCTTAGTGTTGGAGACCTTGACT	AACCTTAGTGTTGGAGACCTTGACA
VGIIa	VGIIa-45211	hypothetical protein	A/C	CCCAGCAACCTTGATCTGGA	AGCTGCTCTAAGAGACACATCATCA	AGCTGCTCTAAGAGACACATCATCC
VGIIb	VGIIb-502129	not annotated	G/A	AATCGCTCGTCCTCATATGACA	GTAGGCGGTGGGATAAGGTG	GGTAGGCGGTGGGATAAGGTA
VGIIc	VGIIc-257655	non-coding region	C/T	CGTTAATTTGGTTGTTTGACAACCT	AGCAACTCACGCAGAAACAGAC	GAGCAACTCACGCAGAAACAGAT
VGIII	VGIII-MPD198	*MPD1 (198)*	T/A	TGACATTGGGACAGTCTGCAAT	ACTGCTGCTTCTCCCGTTGT	CTGCTGCTTCTCCCGTTGA
VGIV	VGIV-MPD423	*MPD1 (423)*	A/C	ACCCAGTCATTAACCTTAGTGTTGGA	CTCGTTCGTCAAYCACGTTAGA	TCGTTCGTCAAYCACGTTAGC

### MAMA design for VGIIa, VGIIb, and VGIIc subtypes

Whole genome sequence typing (WGST) analysis of 20 *C. gattii* strains from a previous study revealed canonical SNPs specific for each of the VGII a, b and c subtypes (n = 2720, 3547, and 3819, respectively) [9]. In order to minimize interference of adjacent mutations with primer design, the genotype-specific SNPs were sorted according to nearest neighboring mismatch within the sequence alignment; in short, the SNPs with the most-conserved flanking regions were the top candidates for assay design. Sequence from the R265 strain reference genome [GenBank: CH408164] [2] surrounding the genotype-specific SNPs was used for assay design. SYBR MAMA primers were designed using the same criteria as previously described for the MLST MAMA (Table 1).

### Isolate selection

Initially, assays were validated with genomic DNA extracted from 57 *C. gattii* strains of North American origin and some historical isolates. The panel of isolates including: 13 VGIIa, 4 VGIIb, and 24 VGIIc, and 8 each of VGI and VGIII, was analyzed using each of the assays (Table 2). All DNAs were genotyped by MLST prior to screening. Further validation of the assays was accomplished by employing a more diverse isolate collection of 55 strains including isolates of international origin; this panel was comprised of 10 VGI, 10 VGIIa, 9 VGIIb, 8 VGIIc, 8 VGIII, and 10 VGIV molecular types (Table 3). The strains came from a variety of environmental, human and animal sources, including cats, a dog, an alpaca, a porpoise, a sheep and a cow.

**Table 2:** C. gattii strains for initial assay validation

**Isolate ID**	**MLST**	**Year**	**Geographic origin**	**Source**
B7488	VGI	2009	Oregon	Human
B7496	VGI	2009	Hawaii	Dolphin
B8551	VGI	2010	Oregon	Human
B8852	VGI	2010	Oregon	Human
B8886	VGI	2010	Oregon	Soil
B8887	VGI	2010	Oregon	Soil
B8990	VGI	2010	California	Human
B9009	VGI	2011	Washington	Human
B6864	VGIIa	2004	Oregon	Human
B7395	VGIIa	2008	Washington	Dog
B7422	VGIIa	2009	Oregon	Cat
B7436	VGIIa	2009	California	Alpaca
B7467	VGIIa	2009	Oregon	Porpoise
B8555	VGIIa	2006	Washington	Human
B8577	VGIIa	2009	British Columbia	Soil
B8793	VGIIa	2010	Oregon	Canine
B8849	VGIIa	2010	Oregon	Environmental
CA-1014	VGIIa	unknown	California	Human
CBS-7750	VGIIa	1990	California	Environmental
ICB-107	VGIIa	unknown	Brazil	Human
NIH-444	VGIIa	1972	Washington	Human
B7394	VGIIb	2008	Washington	Cat
B7735	VGIIb	2009	Oregon	Human
B8554	VGIIb	2010	Oregon	Dog
B8828	VGIIb	2010	Washington	Porpoise
B6863	VGIIc	2005	Oregon	Human
B7390	VGIIc	2008	Idaho	Human
B7432	VGIIc	2009	Oregon	Human
B7434	VGIIc	2008	Oregon	Human
B7466	VGIIc	2008	Oregon	Cat
B7491	VGIIc	2009	Oregon	Human
B7493	VGIIc	2009	Oregon	Sheep
B7641	VGIIc	2008	Oregon	Cat
B7737	VGIIc	2009	Oregon	Human
B7765	VGIIc	2009	Oregon	Dog
B8210	VGIIc	2008	Oregon	Human
B8214	VGIIc	2009	Oregon	Human
B8510	VGIIc	2009	Oregon	Human
B8549	VGIIc	unknown	Oregon	Human
B8552	VGIIc	unknown	Oregon	Human
B8571	VGIIc	2009	Washington	Human
B8788	VGIIc	2010	Oregon	Human
B8798	VGIIc	2005	Oregon	Human
B8821	VGIIc	2010	Oregon	Human
B8825	VGIIc	2009	Oregon	Human
B8833	VGIIc	2010	Oregon	Cat
B8838	VGIIc	2010	Washington	Human
B8843	VGIIc	2010	Oregon	Human
B8853	VGIIc	2010	Oregon	Cat
B7415	VGIII	2009	California	Alpaca
B7495	VGIII	2009	California	Human
B8212	VGIII	2007	Oregon	Human
B8260	VGIII	2009	Washington	Cat
B8262	VGIII	1992	California	Human
B8516/B8616	VGIII	2009	Oregon	Cat
B9143	VGIII	2011	California	Human
B9146	VGIII	2011	California	Human

**Table 3:** C. gattii strains for additional assay validation

**Culture collection ID**	**Geographic origin**	**Sample type**	**MLST**	**Year of isolation**
B4501	Australia	Human	VGI	unknown
B4503	Australia	Human	VGI	unknown
B4504	Australia	Human	VGI	unknown
B4516	Australia	Human	VGI	unknown
B5765	India	Environmental	VGI	unknown
B9018	California	Human	VGI	2011
B9019	New Mexico	Human	VGI	2011
B9021	Rhode Island	Human	VGI	2011
B9142	Georgia	Human	VGI	2011
B9149	California	Human	VGI	2011
B8508	Oregon	Human	VGIIa	2009
B8512	Oregon	Alpaca	VGIIa	2009
B8558	Washington	Human	VGIIa	2010
B8561	Washington	Human	VGIIa	2010
B8563	Washington	Human	VGIIa	2010
B8567	Washington	Dog	VGIIa	2010
B8854	Washington	Human	VGIIa	2010
B8889	Oregon	Environmental	VGIIa	2010
B9077	Washington	Environmental	VGIIa	2011
B9296	British Columbia	Environmental	VGIIa	2011
B8211	Oregon	Human	VGIIb	2009
B8966	Oregon	Horse	VGIIb	2010
B9076	Washington	Environmental	VGIIb	2011
B9157	Washington	Horse	VGIIb	2011
B9170	Washington	Porpoise	VGIIb	2011
B9234	Washington	Cat	VGIIb	2011
B9290	British Columbia	Cat	VGIIb	2011
B9241	Oregon	Human	VGIIb	2011
B9428	Washington	Cat	VGIIb	2012
B9159	Washington	Sheep	VGIIc	2011
B9227	Oregon	Cat	VGIIc	2011
B9235	Oregon	Human	VGIIc	2011
B9244	Oregon	Human	VGIIc	2011
B9245	Oregon	Human	VGIIc	2011
B9295	British Columbia	Environmental	VGIIc	2011
B9302	Oregon	Environmental	VGIIc	2011
B9374	Oregon	Human	VGIIc	2011
B8965	New Mexico	Human	VGIII	2010
B9148	California	Human	VGIII	2011
B9151	Michigan	Human	VGIII	2011
B9163	New Mexico	Human	VGIII	2011
B9237	New Mexico	Cat	VGIII	2011
B9372	California	Cow	VGIII	2011
B9422	Oregon	Cat	VGIII	2012
B9430	Alaska	Cat	VGIII	2012
B7238	Botswana	Human	VGIV	2005
B7240	Botswana	Human	VGIV	2005
B7243	Botswana	Human	VGIV	2005
B7247	Botswana	Human	VGIV	2005
B7249	Botswana	Human	VGIV	2005
B7260	Botswana	Human	VGIV	2006
B7262	Botswana	Human	VGIV	2006
B7263	Botswana	Human	VGIV	2006
B7264	Botswana	Human	VGIV	2006
B7265	Botswana	Human	VGIV	2006

### Isolate culturing and DNA extraction

Isolates were grown on Yeast Peptone Glucose (YPD) agar plus 0.5% NaCl at 37°C for 24 hours; and DNA was prepared using an UltraClean DNA Isolation Kit as described by the manufacturer, with some modifications (MO BIO Laboratories, Carlsbad, CA). Briefly, ~0.5 grams of microbial cells were suspended in lysis solution in a MicroBead tube and heated to 65°C for 15 minutes to increase lysis efficiency. The MicroBead tube was then secured horizontally using the MO BIO vortex adapter tube holder (MO BIO Laboratories, Carlsbad, CA) and vortexed at maximum speed for 10 minutes; post cell lysis, microtubes were immediately placed on ice for 5 minutes. After the lysis steps, DNA extraction was completed per manufacturer’s instructions. DNA was stored at −20°C.

### Real-time PCR

Real-time PCR was performed on the ABI 7900HT real-time PCR System (Life Technologies, Carlsbad, CA). Reactions for both perfect match and mismatch primer sets were conducted in separate wells of a 384-well optical plate, and reactions for each primer set were run in triplicate. Reactions were 10 μL total volume composed of 1X Platinum SYBR Green qPCR SuperMix-UDG with ROX (Invitrogen, Grand Island, NY), 200 nM each of forward and reverse primers, and 1 μL DNA extract (diluted 1:10). Reactions were incubated for 3 min at 50°C for UDG digest followed by 3 min at 95°C for Taq polymerase activation. PCR consisted of 45 cycles of 15 s at 95°C for denaturation followed by 1 min at 60°C annealing and extension. Dissociation of PCR product was performed for 15 sec at 95°C, 15 sec at 60°C and 15 sec at 95°C as a quality assurance step to inspect reactions for primer-dimer. Dissociation curves were not used for isolate genotyping, rather to ensure amplification was specific for the targeted sequence and to preclude non-specific amplification associated with the ability of SYBR Green chemistry to bind any double-stranded DNA. Data were analyzed in Sequence Detection Systems 2.3 software (Life Technologies, Carlsbad, CA) for calculation of cycle threshold (Ct) values and interpretation of dissociation curves.

**Figure 1:**
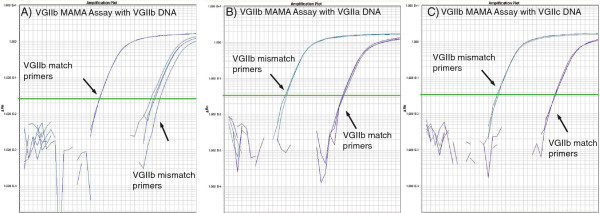
**VGIIb MAMA plots with VGII DNA show the specificity of VGIIb MAMA for VGIIb DNA. (A)** The VGIIb match primers amplify VGIIb DNA efficiently and yield a lower Ct value than the VGIIb mismatch primers, resulting in a VGIIb genotype call. **(B)** The VGIIb mismatch primers amplify VGIIa DNA more efficiently than the VGIIb match primers, resulting in a non-VGIIb genotype call. **(C)** VGIIb mismatch primers amplify VGIIc DNA more efficiently than the VGIIb match primers, again resulting in a non-VGIIb genotype call.

For MAMA results, the perfect match primer set will amplify earlier and yield the lowest Ct value, corresponding to the SNP genotype of the isolate; secondary delayed amplification plots with a higher Ct value, if present, are due to mismatch priming (Figure 1). An algorithm for genotype calling was implemented to expedite data analysis. The delta Ct value was calculated by subtracting the match primer mean Ct from the mismatch primer mean Ct. If the mismatch priming fails to yield a Ct value because it is beyond the instrument range, a Ct value = 40 is assigned in order to calculate a ΔCt.

$$\Delta \mathrm{Ct}=\left(\mathrm{mismatch}\;\mathrm{mean}\;\mathrm{Ct}\right)–\left(\mathrm{perfect}\;\mathrm{match}\;\mathrm{mean}\;\mathrm{Ct}\right)$$

A negative ΔCt value indicates a mismatch allele, whereas a positive ΔCt indicates a match allele. A stringent threshold of |ΔCt| ≥ 3.3, approximately equivalent to one log_10_ difference in the dynamic range, was established to ensure accuracy of allele calls. If |ΔCt| < 3.3 is below the stringent threshold, this could result in an inaccurate genotype call. In this case, it is advisable to re-screen the sample across the failed assays.

Sensitivity and specificity of the assay panel were calculated as well as concordance with the known MLST type as determined by sequencing the MLST house keeping genes. Assay repeatability and reproducibility were tested by screening nine replicate reactions with the matching primer sets and DNA for each assay on three separate days. The lower limit of detection for each assay and its matching template pair was tested. Each matching template and assay pair was tested using six log_10_ serial dilutions of a single template DNA, starting with 0.5 ng/μl. Template DNA was quantified in triplicate by NanoDrop 3300 fluorospectrometer (NanoDrop Technologies, Wilmington, DE) using Quant-iT PicoGreen dsDNA Reagent (Life Technologies, Carlsbad, CA), according to manufacturer’s instructions. Real-time PCR reactions were performed in triplicate for each dilution.

## Results

Initial validation revealed the assay panel was 100% sensitive; each assay appropriately identified the known isolate genotypes. The ΔCt values for our validation panel confirmed the stringent threshold ΔCt = 3.3 sufficient to discriminate the genotypes. In addition, the assay panel was 100% specific; no cross reactivity occurred between assays and non-matching genotypes. Further validation of the assay panel with additional strains revealed 100% sensitivity and specificity. A total of 112 strains were screened across the MLST assay panel and 100% sensitivity and specificity was observed (Table 4). A total of 68 previously genotyped strains were screened across the VGII subtyping assay panel with 100% sensitivity and specificity (Table 5). The assay coefficients of variation ranged from 0.22% to 4.33% indicating high assay repeatability and reproducibility within and between runs (Table 6). The assays were designed for genotyping of DNA from known C. gattii isolates, and are not validated for application to clinical specimens; they were able to detect DNA concentrations as low as 0.5 pg/μl (Table 7).

**Table 4:** MLST SYBR MAMA Ct values and genotype assignments for VGI-VGIV

		**VGI_MPD471**				**VGII_MPD495**				**VGIII_MPD198**				**VGIV_MPD423**				
**Isolate ID**	**Strain type via MLST**	**VGI Ct Mean**	**non-VGI Ct Mean**	**Delta Ct**	**Type call via assay**	**VGII Ct Mean**	**non-VGII Ct Mean**	**Delta Ct**	**Type call via assay**	**VGIII Ct Mean**	**non-VGIII Ct Mean**	**Delta Ct**	**Type call via assay**	**VGIV Ct Mean**	**non-VGIV Ct Mean**	**Delta Ct**	**Type call via assay**	**Final Call**
B7488	VGI	17.0	29.0	11.9	VGI	37.4	17.7	−19.7	non-VGII	28.4	14.9	−13.5	non-VGIII	32.4	16.3	−16.1	non-VGIV	**VGI**
B7496	VGI	18.2	28.0	9.8	VGI	35.3	19.0	−16.3	non-VGII	24.5	16.4	−8.1	non-VGIII	31.7	17.9	−13.8	non-VGIV	**VGI**
B8551	VGI	17.3	29.6	12.3	VGI	36.2	17.9	−18.3	non-VGII	28.7	15.3	−13.4	non-VGIII	39.0	16.7	−22.3	non-VGIV	**VGI**
B8852	VGI	21.1	30.9	9.8	VGI	36.5	21.9	−14.6	non-VGII	27.8	19.1	−8.8	non-VGIII	32.0	20.6	−11.4	non-VGIV	**VGI**
B8886	VGI	18.9	29.2	10.3	VGI	38.1	19.3	−18.8	non-VGII	26.7	16.4	−10.3	non-VGIII	32.3	17.9	−14.4	non-VGIV	**VGI**
B8887	VGI	15.9	28.3	12.4	VGI	23.6	15.5	−8.1	non-VGII	33.6	16.2	−17.4	non-VGIII	34.1	15.5	−18.7	non-VGIV	**VGI**
B8990	VGI	18.8	30.9	12.1	VGI	37.2	20.1	−17.1	non-VGII	31.3	16.9	−14.3	non-VGIII	40.0	19.3	−20.7	non-VGIV	**VGI**
B9009	VGI	21.6	31.0	9.4	VGI	36.5	23.1	−13.4	non-VGII	28.6	19.4	−9.2	non-VGIII	40.0	21.1	−18.9	non-VGIV	**VGI**
B4501	VGI	16.1	26.7	10.6	VGI	30.5	18.1	−12.4	non-VGII	30.6	17.3	−13.3	non-VGIII	29.4	16.4	−13.0	non-VGIV	**VGI**
B4503	VGI	15.9	27.2	11.2	VGI	32.7	18.6	−14.1	non-VGII	33.8	17.9	−15.9	non-VGIII	28.7	16.1	−12.6	non-VGIV	**VGI**
B4504	VGI	15.6	27.2	11.5	VGI	33.1	18.1	−15.1	non-VGII	33.9	17.4	−16.4	non-VGIII	28.7	15.8	−13.0	non-VGIV	**VGI**
B4516	VGI	15.3	26.8	11.5	VGI	31.5	17.6	−13.9	non-VGII	33.4	16.8	−16.6	non-VGIII	29.7	15.3	−14.3	non-VGIV	**VGI**
B5765	VGI	17.2	28.0	10.8	VGI	32.8	19.7	−13.0	non-VGII	34.4	19.2	−15.2	non-VGIII	29.0	16.3	−12.7	non-VGIV	**VGI**
B9018	VGI	17.7	30.0	12.3	VGI	34.6	17.9	−16.7	non-VGII	31.8	18.6	−13.2	non-VGIII	35.0	18.3	−16.8	non-VGIV	**VGI**
B9019	VGI	16.9	26.1	9.2	VGI	35.4	16.7	−18.7	non-VGII	34.9	16.7	−18.2	non-VGIII	30.5	16.8	−13.7	non-VGIV	**VGI**
B9021	VGI	21.4	32.9	11.5	VGI	33.4	19.9	−13.5	non-VGII	32.7	20.5	−12.2	non-VGIII	35.5	20.4	−15.2	non-VGIV	**VGI**
B9142	VGI	16.0	26.3	10.3	VGI	27.8	15.9	−11.9	non-VGII	32.7	16.5	−16.2	non-VGIII	31.7	16.6	−15.1	non-VGIV	**VGI**
B9149	VGI	17.7	26.8	9.1	VGI	28.5	17.5	−11.0	non-VGII	28.5	18.2	−10.3	non-VGIII	31.0	18.3	−12.6	non-VGIV	**VGI**
B6864	VGIIa	27.8	17.5	−10.3	non-VGI	19.3	33.1	13.8	VGII	34.7	19.7	−15.0	non-VGIII	40.0	16.1	−23.9	non-VGIV	**VGII**
B7395	VGIIa	28.9	18.8	−10.1	non-VGI	21.3	32.6	11.3	VGII	40.0	19.2	19.2	non-VGIII	40.0	18.8	−21.2	non-VGIV	**VGII**
B7422	VGIIa	27.4	17.4	−10.0	non-VGI	19.5	32.3	12.8	VGII	35.4	19.1	−16.3	non-VGIII	40.0	15.6	−24.4	non-VGIV	**VGII**
B7436	VGIIa	27.8	17.9	−9.9	non-VGI	20.7	35.4	14.7	VGII	36.5	16.9	−19.6	non-VGIII	40.0	15.6	−24.4	non-VGIV	**VGII**
B7467	VGIIa	30.9	20.7	−10.1	non-VGI	22.7	32.7	9.9	VGII	37.7	23.4	−14.2	non-VGIII	40.0	19.1	−20.9	non-VGIV	**VGII**
B8555	VGIIa	27.9	17.7	−10.2	non-VGI	19.7	32.1	12.4	VGII	34.6	20.8	−13.8	non-VGIII	40.0	16.6	−23.4	non-VGIV	**VGII**
B8577	VGIIa	31.1	20.9	−10.2	non-VGI	21.8	34.1	12.3	VGII	33.1	23.4	−9.8	non-VGIII	40.0	19.8	−20.2	non-VGIV	**VGII**
B8793	VGIIa	27.4	17.4	−10.0	non-VGI	18.9	32.6	13.7	VGII	39.0	24.9	−14.1	non-VGIII	40.0	16.3	−23.7	non-VGIV	**VGII**
B8849	VGIIa	28.9	18.7	−10.1	non-VGI	22.9	35.1	12.2	VGII	36.0	22.7	−13.3	non-VGIII	40.0	18.4	−21.6	non-VGIV	**VGII**
CA-1014	VGIIa	20.4	11.6	−8.8	non-VGI	13.6	32.4	18.9	VGII	31.1	12.8	−18.3	non-VGIII	40.0	11.0	−29.0	non-VGIV	**VGII**
CBS-7750	VGIIa	27.2	17.3	−9.9	non-VGI	18.8	33.1	14.3	VGII	38.0	25.5	−12.5	non-VGIII	40.0	15.8	−24.2	non-VGIV	**VGII**
ICB-107	VGIIa	28.1	18.2	−9.9	non-VGI	20.0	34.7	14.8	VGII	37.5	25.4	−12.1	non-VGIII	40.0	15.6	−24.4	non-VGIV	**VGII**
NIH-444	VGIIa	24.9	14.9	−10.0	non-VGI	17.0	33.2	16.2	VGII	34.9	17.7	−17.2	non-VGIII	40.0	13.3	−26.7	non-VGIV	**VGII**
B8508	VGIIa	23.7	14.8	−8.9	non-VGI	17.4	30.4	13.0	VGII	34.5	16.2	−18.2	non-VGIII	29.1	14.9	−14.2	non-VGIV	**VGII**
B8512	VGIIa	23.5	14.6	−9.0	non-VGI	16.7	30.6	13.9	VGII	31.4	15.7	−15.6	non-VGIII	29.7	14.8	−14.9	non-VGIV	**VGII**
B8558	VGIIa	22.5	13.7	−8.8	non-VGI	15.9	29.9	14.0	VGII	30.6	14.9	−15.7	non-VGIII	30.1	14.3	−15.9	non-VGIV	**VGII**
B8561	VGIIa	26.5	17.7	−8.8	non-VGI	20.3	34.2	14.0	VGII	34.1	19.1	−15.0	non-VGIII	33.2	22.2	−11.0	non-VGIV	**VGII**
B8563	VGIIa	24.4	16.0	−8.4	non-VGI	18.4	32.8	14.4	VGII	32.8	20.4	−12.4	non-VGIII	32.2	17.3	−14.9	non-VGIV	**VGII**
B8567	VGIIa	25.6	17.0	−8.6	non-VGI	19.4	34.1	14.7	VGII	33.8	18.2	−15.6	non-VGIII	35.1	16.8	−18.2	non-VGIV	**VGII**
B8854	VGIIa	24.7	15.8	−8.9	non-VGI	18.1	32.7	14.6	VGII	33.0	17.1	−15.9	non-VGIII	33.2	15.8	−17.4	non-VGIV	**VGII**
B8889	VGIIa	28.0	17.6	−10.4	non-VGI	20.3	33.1	12.7	VGII	33.7	19.1	−14.6	non-VGIII	32.4	17.5	−15.0	non-VGIV	**VGII**
B9077	VGIIa	33.6	17.8	−15.9	non-VGI	15.4	28.6	13.2	VGII	40.0	18.6	−21.5	non-VGIII	40.0	18.6	−21.4	non-VGIV	**VGII**
B9296	VGIIa	27.3	19.8	−7.5	non-VGI	18.6	34.0	15.4	VGII	32.4	20.8	−11.6	non-VGIII	34.9	19.2	−15.7	non-VGIV	**VGII**
B7394	VGIIb	31.9	22.5	−9.5	non-VGI	23.5	33.5	10.0	VGII	33.7	19.3	−14.4	non-VGIII	40.0	20.2	−19.8	non-VGIV	**VGII**
B7735	VGIIb	26.9	17.8	−9.1	non-VGI	18.3	33.3	15.0	VGII	0.0	15.8	15.8	non-VGIII	40.0	15.4	−24.6	non-VGIV	**VGII**
B8554	VGIIb	28.8	18.3	−10.5	non-VGI	20.8	32.2	11.3	VGII	35.5	22.0	−13.4	non-VGIII	40.0	18.3	−21.7	non-VGIV	**VGII**
B8828	VGIIb	28.8	18.5	−10.3	non-VGI	20.7	32.7	11.9	VGII	35.9	19.2	−16.7	non-VGIII	40.0	31.9	−8.1	non-VGIV	**VGII**
B8211	VGIIb	22.9	12.8	−10.1	non-VGI	15.1	30.1	15.1	VGII	33.0	13.9	−19.0	non-VGIII	33.8	12.9	−21.0	non-VGIV	**VGII**
B8966	VGIIb	24.6	15.5	−9.0	non-VGI	17.3	25.9	8.6	VGII	29.3	15.6	−13.7	non-VGIII	28.9	14.7	−14.2	non-VGIV	**VGII**
B9076	VGIIb	40.0	17.5	−22.5	non-VGI	17.1	27.5	10.5	VGII	40.0	18.4	−21.6	non-VGIII	30.6	18.0	−12.6	non-VGIV	**VGII**
B9157	VGIIb	25.4	15.3	−10.2	non-VGI	17.6	29.4	11.9	VGII	31.2	16.1	−15.1	non-VGIII	31.6	16.1	−15.5	non-VGIV	**VGII**
B9170	VGIIb	26.2	16.9	−9.3	non-VGI	17.5	28.7	11.2	VGII	29.5	17.6	−11.9	non-VGIII	31.1	17.7	−13.4	non-VGIV	**VGII**
B9234	VGIIb	24.7	15.0	−9.6	non-VGI	15.4	30.3	14.9	VGII	30.2	15.7	−14.5	non-VGIII	33.3	15.8	−17.5	non-VGIV	**VGII**
B9290	VGIIb	24.8	16.0	−8.8	non-VGI	15.9	34.1	18.2	VGII	30.6	20.8	−9.7	non-VGIII	33.2	16.6	−16.6	non-VGIV	**VGII**
B9241	VGIIb	23.4	13.2	−10.3	non-VGI	15.5	28.0	12.5	VGII	30.0	13.9	−16.0	non-VGIII	34.0	13.5	−20.5	non-VGIV	**VGII**
B9428	VGIIb	25.2	14.4	−10.7	non-VGI	18.7	28.3	9.6	VGII	30.2	15.5	−14.7	non-VGIII	34.1	15.0	−19.1	non-VGIV	**VGII**
B6863	VGIIc	28.9	18.6	−10.2	non-VGI	20.7	34.2	13.5	VGII	33.2	22.7	−10.6	non-VGIII	40.0	18.1	−21.9	non-VGIV	**VGII**
B7390	VGIIc	27.7	18.3	−9.5	non-VGI	19.9	33.9	13.9	VGII	39.5	24.7	−14.8	non-VGIII	40.0	16.9	−23.1	non-VGIV	**VGII**
B7432	VGIIc	28.2	18.3	−9.9	non-VGI	20.0	32.6	12.7	VGII	34.8	18.0	−16.8	non-VGIII	40.0	17.2	−22.8	non-VGIV	**VGII**
B7434	VGIIc	25.6	16.2	−9.4	non-VGI	17.7	34.5	16.8	VGII	34.4	17.9	−16.5	non-VGIII	40.0	13.8	−26.2	non-VGIV	**VGII**
B7466	VGIIc	30.8	20.8	−10.0	non-VGI	22.4	33.6	11.2	VGII	37.4	23.7	−13.7	non-VGIII	40.0	19.5	−20.5	non-VGIV	**VGII**
B7491	VGIIc	26.9	17.3	−9.6	non-VGI	19.2	33.0	13.8	VGII	0.0	16.8	16.8	non-VGIII	40.0	16.7	−23.3	non-VGIV	**VGII**
B7493	VGIIc	27.1	17.4	−9.7	non-VGI	18.6	33.6	15.1	VGII	36.6	20.7	−15.8	non-VGIII	40.0	16.1	−23.9	non-VGIV	**VGII**
B7641	VGIIc	26.0	17.3	−8.7	non-VGI	18.7	32.3	13.7	VGII	34.3	20.0	−14.3	non-VGIII	40.0	15.6	−24.4	non-VGIV	**VGII**
B7737	VGIIc	28.0	18.5	−9.6	non-VGI	20.1	34.3	14.2	VGII	37.0	23.0	−14.0	non-VGIII	40.0	18.0	−22.0	non-VGIV	**VGII**
B7765	VGIIc	22.5	13.0	−9.5	non-VGI	14.5	34.1	19.6	VGII	33.1	23.4	−9.7	non-VGIII	40.0	12.9	−27.1	non-VGIV	**VGII**
B8210	VGIIc	27.8	18.1	−9.7	non-VGI	19.6	33.3	13.7	VGII	33.0	19.4	−13.5	non-VGIII	40.0	16.8	−23.2	non-VGIV	**VGII**
B8214	VGIIc	27.1	17.7	−9.5	non-VGI	19.8	34.9	15.1	VGII	34.1	20.1	−14.0	non-VGIII	40.0	16.1	−23.9	non-VGIV	**VGII**
B8510	VGIIc	26.8	17.6	−9.2	non-VGI	18.8	33.2	14.5	VGII	35.2	19.1	−16.1	non-VGIII	40.0	15.6	−24.4	non-VGIV	**VGII**
B8549	VGIIc	26.8	16.2	−10.6	non-VGI	18.7	33.5	14.8	VGII	37.4	20.5	−16.9	non-VGIII	40.0	29.6	−10.4	non-VGIV	**VGII**
B8552	VGIIc	27.1	17.0	−10.1	non-VGI	18.6	33.2	14.6	VGII	34.3	19.7	−14.6	non-VGIII	40.0	16.6	−23.4	non-VGIV	**VGII**
B8571	VGIIc	28.8	19.4	−9.4	non-VGI	21.5	33.4	11.9	VGII	34.5	22.8	−11.8	non-VGIII	40.0	19.5	−20.5	non-VGIV	**VGII**
B8788	VGIIc	26.0	16.0	−10.0	non-VGI	18.5	29.5	11.0	VGII	38.0	20.4	−17.6	non-VGIII	40.0	16.6	−23.4	non-VGIV	**VGII**
B8798	VGIIc	36.0	24.7	−11.4	non-VGI	26.5	33.3	6.8	VGII	37.2	19.2	−18.0	non-VGIII	40.0	22.5	−17.5	non-VGIV	**VGII**
B8821	VGIIc	30.5	20.5	−10.0	non-VGI	22.3	33.0	10.7	VGII	37.0	29.0	−8.0	non-VGIII	40.0	18.7	−21.3	non-VGIV	**VGII**
B8825	VGIIc	27.4	17.8	−9.6	non-VGI	19.6	33.7	14.1	VGII	36.0	20.5	−15.5	non-VGIII	40.0	17.5	−22.5	non-VGIV	**VGII**
B8833	VGIIc	29.2	20.7	−8.6	non-VGI	19.5	33.4	13.9	VGII	35.4	19.6	−15.8	non-VGIII	40.0	15.5	−24.5	non-VGIV	**VGII**
B8838	VGIIc	29.2	19.1	−10.1	non-VGI	21.5	32.8	11.3	VGII	32.9	22.3	−10.6	non-VGIII	40.0	18.5	−21.5	non-VGIV	**VGII**
B8843	VGIIc	29.5	19.4	−10.1	non-VGI	21.5	33.7	12.2	VGII	37.5	22.1	−15.4	non-VGIII	40.0	19.1	−20.9	non-VGIV	**VGII**
B8853	VGIIc	33.3	23.1	−10.2	non-VGI	24.8	33.7	8.9	VGII	34.2	27.8	−6.4	non-VGIII	40.0	21.5	−18.5	non-VGIV	**VGII**
B9159	VGIIc	29.6	17.5	−12.1	non-VGI	19.1	29.9	10.7	VGII	40.0	26.0	−14.0	non-VGIII	40.0	18.0	−22.0	non-VGIV	**VGII**
B9227	VGIIc	24.4	15.3	−9.1	non-VGI	15.5	28.1	12.6	VGII	27.9	16.1	−11.9	non-VGIII	31.0	16.3	−14.7	non-VGIV	**VGII**
B9235	VGIIc	24.6	15.1	−9.5	non-VGI	15.3	28.9	13.7	VGII	29.2	16.4	−12.7	non-VGIII	31.2	15.9	−15.3	non-VGIV	**VGII**
B9244	VGIIc	27.3	18.4	−8.9	non-VGI	18.5	31.8	13.3	VGII	28.2	21.0	−7.2	non-VGIII	30.6	18.8	−11.8	non-VGIV	**VGII**
B9245	VGIIc	26.8	17.9	−8.9	non-VGI	18.0	33.5	15.5	VGII	31.2	19.3	−11.9	non-VGIII	34.2	18.5	−15.6	non-VGIV	**VGII**
B9295	VGIIc	28.6	19.5	−9.1	non-VGI	19.9	40.0	20.1	VGII	33.6	25.5	−8.1	non-VGIII	34.4	20.3	−14.2	non-VGIV	**VGII**
B9302	VGIIc	24.6	14.1	−10.5	non-VGI	16.9	26.7	9.8	VGII	28.8	15.1	−13.7	non-VGIII	31.5	14.1	−17.3	non-VGIV	**VGII**
B9374	VGIIc	24.8	14.2	−10.6	non-VGI	18.2	27.3	9.1	VGII	29.1	15.2	−13.9	non-VGIII	32.8	14.4	−18.4	non-VGIV	**VGII**
B7415	VGIII	26.8	15.9	−10.9	non-VGI	35.0	17.7	−17.3	non-VGII	12.4	27.1	14.7	VGIII	30.9	15.9	−15.0	non-VGIV	**VGIII**
B7495	VGIII	28.1	18.0	−10.1	non-VGI	36.1	18.8	−17.3	non-VGII	14.1	30.1	16.0	VGIII	31.8	17.6	−14.2	non-VGIV	**VGIII**
B8212	VGIII	26.0	15.7	−10.3	non-VGI	35.3	17.0	−18.3	non-VGII	12.4	28.5	16.1	VGIII	32.5	15.6	−16.9	non-VGIV	**VGIII**
B8260	VGIII	29.6	19.6	−10.0	non-VGI	36.7	20.8	−15.9	non-VGII	15.9	30.7	14.8	VGIII	36.0	19.1	−16.9	non-VGIV	**VGIII**
B8262	VGIII	27.2	17.2	−10.0	non-VGI	33.8	18.3	−15.5	non-VGII	13.5	30.0	16.4	VGIII	40.0	16.9	−23.1	non-VGIV	**VGIII**
B8516/B8616	VGIII	28.4	18.5	−9.9	non-VGI	37.8	19.5	−18.3	non-VGII	14.6	29.1	14.5	VGIII	31.8	18.0	−13.8	non-VGIV	**VGIII**
B9143	VGIII	28.6	18.3	−10.3	non-VGI	38.3	19.6	−18.7	non-VGII	14.5	30.2	15.7	VGIII	33.3	18.0	−15.3	non-VGIV	**VGIII**
B9146	VGIII	30.3	19.5	−10.8	non-VGI	38.5	21.2	−17.3	non-VGII	15.8	30.1	14.3	VGIII	31.2	19.3	−11.9	non-VGIV	**VGIII**
B8965	VGIII	26.2	16.8	−9.4	non-VGI	30.6	17.1	−13.5	non-VGII	16.1	30.6	14.5	VGIII	35.0	17.4	−17.6	non-VGIV	**VGIII**
B9148	VGIII	26.0	16.6	−9.4	non-VGI	31.0	16.6	−14.4	non-VGII	15.9	30.6	14.7	VGIII	32.8	17.4	−15.4	non-VGIV	**VGIII**
B9151	VGIII	25.7	16.5	−9.3	non-VGI	30.7	16.2	−14.4	non-VGII	15.4	30.3	14.9	VGIII	34.9	18.0	−17.0	non-VGIV	**VGIII**
B9163	VGIII	26.9	17.5	−9.4	non-VGI	29.8	17.3	−12.5	non-VGII	16.9	29.7	12.8	VGIII	33.4	18.0	−15.4	non-VGIV	**VGIII**
B9237	VGIII	26.7	17.9	−8.9	non-VGI	31.6	17.4	−14.2	non-VGII	17.3	35.0	17.7	VGIII	38.1	19.3	−18.9	non-VGIV	**VGIII**
B9372	VGIII	23.5	12.7	−10.9	non-VGI	29.3	13.1	−16.1	non-VGII	14.8	27.4	12.6	VGIII	32.6	13.0	−19.6	non-VGIV	**VGIII**
B9422	VGIII	23.9	12.8	−11.1	non-VGI	28.9	12.9	−15.9	non-VGII	14.6	26.8	12.2	VGIII	33.0	13.3	−19.7	non-VGIV	**VGIII**
B9430	VGIII	23.5	12.9	−10.6	non-VGI	30.1	13.4	−16.8	non-VGII	15.1	28.5	13.4	VGIII	35.5	13.4	−22.0	non-VGIV	**VGIII**
B7238	VGIV	25.2	16.4	−8.8	non-VGI	33.2	18.5	−14.7	non-VGII	34.6	17.9	−16.7	non-VGIII	16.3	27.4	11.1	VGIV	**VGIV**
B7240	VGIV	25.8	17.1	−8.8	non-VGI	33.9	19.5	−14.5	non-VGII	34.2	18.5	−15.7	non-VGIII	17.0	28.8	11.8	VGIV	**VGIV**
B7243	VGIV	26.1	17.3	−8.8	non-VGI	32.0	19.6	−12.4	non-VGII	32.3	18.7	−13.6	non-VGIII	16.8	27.1	10.2	VGIV	**VGIV**
B7247	VGIV	25.6	16.5	−9.1	non-VGI	33.4	19.2	−14.2	non-VGII	32.0	18.1	−13.9	non-VGIII	16.3	28.4	12.1	VGIV	**VGIV**
B7249	VGIV	23.4	14.8	−8.6	non-VGI	31.6	16.7	−14.9	non-VGII	32.6	16.0	−16.6	non-VGIII	14.5	31.1	16.5	VGIV	**VGIV**
B7260	VGIV	26.0	16.5	−9.4	non-VGI	30.9	18.0	−13.0	non-VGII	34.2	17.4	−16.8	non-VGIII	15.7	27.0	11.2	VGIV	**VGIV**
B7262	VGIV	26.3	16.8	−9.5	non-VGI	31.4	18.7	−12.7	non-VGII	33.4	18.0	−15.4	non-VGIII	15.8	27.5	11.6	VGIV	**VGIV**
B7263	VGIV	24.5	15.7	−8.9	non-VGI	33.1	17.9	−15.3	non-VGII	37.3	17.0	−20.3	non-VGIII	15.8	28.0	12.2	VGIV	**VGIV**
B7264	VGIV	24.4	15.0	−9.4	non-VGI	31.2	16.9	−14.3	non-VGII	30.6	16.0	−14.6	non-VGIII	14.8	26.8	12.0	VGIV	**VGIV**
B7265	VGIV	27.5	17.3	−10.2	non-VGI	34.1	19.6	−14.5	non-VGII	32.1	18.8	−13.3	non-VGIII	16.9	28.8	11.9	VGIV	**VGIV**

**Table 5:** VGII subtyping SYBR MAMA Ct values and genotype assignments for VGIIa,b,c

		**VGIIa_Assay_45211**				**VGIIb_Assay_502129**				**VGIIc_Assay_257655**				
**Isolate ID**	**Strain type via MLST**	**VGIIa Ct Mean**	**non-VGIIa Ct Mean**	**Delta Ct**	**Type call via assay**	**VGIIb Ct Mean**	**non-VGIIb Ct Mean**	**Delta Ct**	**Type call via assay**	**VGIIc Ct Mean**	**non-VGIIc Ct Mean**	**Delta Ct**	**Type call via assay**	**Final Call**
B6864	VGIIa	17.2	30.5	13.3	VGIIa	31.0	17.5	−13.5	non-VGIIb	40.0	27.8	−12.2	non-VGIIc	**VGIIa**
B7395	VGIIa	19.8	33.5	13.7	VGIIa	33.1	20.3	−12.9	non-VGIIb	40.0	30.6	−9.4	non-VGIIc	**VGIIa**
B7422	VGIIa	18.3	33.6	15.4	VGIIa	26.4	17.6	−8.8	non-VGIIb	39.2	28.6	−10.6	non-VGIIc	**VGIIa**
B7436	VGIIa	18.6	31.7	13.1	VGIIa	30.1	17.0	−13.2	non-VGIIb	38.0	29.1	−8.9	non-VGIIc	**VGIIa**
B7467	VGIIa	20.5	37.3	16.8	VGIIa	35.1	20.3	−14.7	non-VGIIb	40.0	30.9	−9.1	non-VGIIc	**VGIIa**
B8555	VGIIa	17.1	31.2	14.1	VGIIa	30.3	17.5	−12.8	non-VGIIb	40.0	27.7	−12.3	non-VGIIc	**VGIIa**
B8577	VGIIa	20.8	36.8	16.0	VGIIa	32.8	20.8	−12.1	non-VGIIb	40.0	31.4	−8.6	non-VGIIc	**VGIIa**
B8793	VGIIa	15.1	29.8	14.7	VGIIa	30.7	18.6	−12.1	non-VGIIb	40.0	29.8	−10.2	non-VGIIc	**VGIIa**
B8849	VGIIa	19.8	34.4	14.6	VGIIa	33.6	20.2	−13.4	non-VGIIb	40.0	30.6	−9.4	non-VGIIc	**VGIIa**
CA-1014	VGIIa	13.1	27.3	14.2	VGIIa	27.0	14.0	−13.0	non-VGIIb	34.9	24.2	−10.7	non-VGIIc	**VGIIa**
CBS-7750	VGIIa	21.8	32.2	10.4	VGIIa	33.4	21.5	−11.9	non-VGIIb	40.0	34.1	−5.9	non-VGIIc	**VGIIa**
ICB-107	VGIIa	21.8	33.6	11.8	VGIIa	33.2	21.2	−12.0	non-VGIIb	40.0	33.8	−6.2	non-VGIIc	**VGIIa**
NIH-444	VGIIa	14.8	27.3	12.5	VGIIa	28.5	15.3	−13.1	non-VGIIb	36.1	25.7	−10.3	non-VGIIc	**VGIIa**
B8508	VGIIa	17.0	27.8	10.8	VGIIa	26.5	17.3	−9.2	non-VGIIb	31.7	22.7	−9.1	non-VGIIc	**VGIIa**
B8512	VGIIa	17.6	28.1	10.4	VGIIa	26.3	18.0	−8.3	non-VGIIb	33.2	24.2	−9.0	non-VGIIc	**VGIIa**
B8558	VGIIa	16.3	24.8	8.5	VGIIa	27.3	15.3	−12.0	non-VGIIb	29.4	20.0	−9.4	non-VGIIc	**VGIIa**
B8561	VGIIa	15.8	27.5	11.8	VGIIa	25.0	16.9	−8.1	non-VGIIb	33.4	23.2	−10.2	non-VGIIc	**VGIIa**
B8563	VGIIa	14.5	27.3	12.8	VGIIa	23.9	15.6	−8.3	non-VGIIb	31.7	21.7	−10.0	non-VGIIc	**VGIIa**
B8567	VGIIa	15.0	36.2	21.2	VGIIa	24.5	16.0	−8.5	non-VGIIb	31.8	22.2	−9.5	non-VGIIc	**VGIIa**
B8854	VGIIa	14.7	26.7	12.0	VGIIa	24.1	15.1	−9.0	non-VGIIb	31.4	22.2	−9.2	non-VGIIc	**VGIIa**
B8889	VGIIa	17.0	28.1	11.0	VGIIa	25.9	17.3	−8.7	non-VGIIb	33.2	23.8	−9.4	non-VGIIc	**VGIIa**
B9077	VGIIa	16.7	27.8	11.1	VGIIa	25.6	16.7	−9.0	non-VGIIb	32.9	24.4	−8.4	non-VGIIc	**VGIIa**
B9296	VGIIa	17.0	27.5	10.5	VGIIa	25.5	17.3	−8.2	non-VGIIb	32.9	24.8	−8.1	non-VGIIc	**VGIIa**
B7394	VGIIb	40.0	19.0	−21.0	non-VGIIa	17.3	29.6	12.3	VGIIb	40.0	29.0	−11.0	non-VGIIc	**VGIIb**
B7735	VGIIb	31.0	18.3	−12.8	non-VGIIa	18.7	31.3	12.6	VGIIb	38.1	28.9	−9.3	non-VGIIc	**VGIIb**
B8554	VGIIb	32.9	21.2	−11.7	non-VGIIa	22.2	35.0	12.8	VGIIb	40.0	30.4	−9.6	non-VGIIc	**VGIIb**
B8828	VGIIb	31.9	21.1	−10.8	non-VGIIa	19.9	35.1	15.2	VGIIb	40.0	30.5	−9.5	non-VGIIc	**VGIIb**
B8211	VGIIb	27.8	16.9	−10.9	non-VGIIa	17.4	28.8	11.4	VGIIb	32.3	22.3	−10.0	non-VGIIc	**VGIIb**
B8966	VGIIb	26.2	14.7	−11.5	non-VGIIa	16.3	24.1	7.9	VGIIb	31.8	23.2	−8.6	non-VGIIc	**VGIIb**
B9076	VGIIb	30.0	18.8	−11.2	non-VGIIa	19.7	30.9	11.4	VGIIb	39.1	27.0	−12.1	non-VGIIc	**VGIIb**
B9157	VGIIb	29.1	16.6	−12.4	non-VGIIa	15.4	23.8	8.5	VGIIb	30.3	21.3	−9.0	non-VGIIc	**VGIIb**
B9170	VGIIb	26.6	15.4	−11.2	non-VGIIa	16.9	24.8	7.9	VGIIb	31.0	22.7	−8.3	non-VGIIc	**VGIIb**
B9234	VGIIb	26.1	13.9	−12.2	non-VGIIa	15.3	23.8	8.5	VGIIb	30.2	21.2	−9.1	non-VGIIc	**VGIIb**
B9290	VGIIb	26.1	13.8	−12.3	non-VGIIa	15.1	24.5	9.5	VGIIb	30.6	21.2	−9.5	non-VGIIc	**VGIIb**
B9241	VGIIb	26.7	20.2	−6.5	non-VGIIa	14.5	24.0	9.4	VGIIb	30.5	21.4	−9.1	non-VGIIc	**VGIIb**
B9428	VGIIb	27.5	14.8	−12.6	non-VGIIa	16.0	24.3	8.2	VGIIb	32.0	22.4	−9.6	non-VGIIc	**VGIIb**
B6863	VGIIc	31.9	20.3	−11.5	non-VGIIa	33.4	20.2	−13.2	non-VGIIb	27.5	40.0	12.5	VGIIc	**VGIIc**
B7390	VGIIc	32.7	18.9	−13.8	non-VGIIa	31.1	17.9	−13.2	non-VGIIb	25.9	40.0	14.1	VGIIc	**VGIIc**
B7432	VGIIc	40.0	18.5	−21.5	non-VGIIa	30.7	17.6	−13.1	non-VGIIb	25.7	40.0	14.3	VGIIc	**VGIIc**
B7434	VGIIc	27.5	15.5	−12.0	non-VGIIa	28.5	15.4	−13.1	non-VGIIb	23.3	40.0	16.7	VGIIc	**VGIIc**
B7466	VGIIc	31.7	20.8	−10.9	non-VGIIa	33.5	20.6	−12.8	non-VGIIb	28.1	40.0	11.9	VGIIc	**VGIIc**
B7491	VGIIc	28.7	17.4	−11.2	non-VGIIa	30.4	16.9	−13.5	non-VGIIb	24.0	40.0	16.0	VGIIc	**VGIIc**
B7493	VGIIc	28.8	18.3	−10.6	non-VGIIa	31.1	18.0	−13.1	non-VGIIb	25.5	40.0	14.5	VGIIc	**VGIIc**
B7641	VGIIc	29.2	17.2	−12.0	non-VGIIa	30.0	17.2	−12.8	non-VGIIb	24.5	40.0	15.5	VGIIc	**VGIIc**
B7737	VGIIc	32.6	20.1	−12.5	non-VGIIa	30.8	20.5	−10.4	non-VGIIb	28.4	40.0	11.6	VGIIc	**VGIIc**
B7765	VGIIc	32.2	19.3	−12.8	non-VGIIa	32.3	18.9	−13.3	non-VGIIb	27.5	40.0	12.5	VGIIc	**VGIIc**
B8210	VGIIc	29.7	17.6	−12.0	non-VGIIa	30.1	17.4	−12.7	non-VGIIb	25.9	40.0	14.1	VGIIc	**VGIIc**
B8214	VGIIc	30.1	17.5	−12.5	non-VGIIa	30.9	17.5	−13.4	non-VGIIb	26.1	40.0	13.9	VGIIc	**VGIIc**
B8510	VGIIc	29.6	17.5	−12.0	non-VGIIa	31.0	17.3	−13.7	non-VGIIb	24.5	40.0	15.5	VGIIc	**VGIIc**
B8549	VGIIc	29.9	17.7	−12.1	non-VGIIa	31.0	17.8	−13.2	non-VGIIb	24.8	40.0	15.2	VGIIc	**VGIIc**
B8552	VGIIc	29.2	17.1	−12.0	non-VGIIa	30.3	17.2	−13.1	non-VGIIb	24.4	40.0	15.6	VGIIc	**VGIIc**
B8571	VGIIc	33.0	20.3	−12.7	non-VGIIa	32.6	20.2	−12.5	non-VGIIb	28.1	40.0	11.9	VGIIc	**VGIIc**
B8788	VGIIc	29.1	17.3	−11.7	non-VGIIa	30.0	17.2	−12.8	non-VGIIb	25.0	40.0	15.0	VGIIc	**VGIIc**
B8798	VGIIc	36.5	22.8	−13.7	non-VGIIa	34.5	22.2	−12.3	non-VGIIb	31.0	40.0	9.0	VGIIc	**VGIIc**
B8821	VGIIc	37.7	24.5	−13.2	non-VGIIa	37.1	24.4	−12.7	non-VGIIb	33.0	40.0	7.0	VGIIc	**VGIIc**
B8825	VGIIc	29.6	17.7	−11.9	non-VGIIa	30.6	17.7	−12.9	non-VGIIb	25.8	40.0	14.2	VGIIc	**VGIIc**
B8833	VGIIc	29.0	17.0	−12.0	non-VGIIa	30.1	17.0	−13.1	non-VGIIb	25.2	40.0	14.8	VGIIc	**VGIIc**
B8838	VGIIc	32.0	19.5	−12.5	non-VGIIa	32.9	19.3	−13.7	non-VGIIb	28.7	40.0	11.3	VGIIc	**VGIIc**
B8843	VGIIc	32.4	19.9	−12.5	non-VGIIa	33.0	19.5	−13.5	non-VGIIb	28.6	40.0	11.4	VGIIc	**VGIIc**
B8853	VGIIc	32.8	21.5	−11.3	non-VGIIa	36.0	23.4	−12.6	non-VGIIb	33.1	40.0	6.9	VGIIc	**VGIIc**
B9159	VGIIc	27.4	20.3	−7.1	non-VGIIa	25.8	16.7	−9.1	non-VGIIb	20.5	34.5	14.0	VGIIc	**VGIIc**
B9227	VGIIc	25.6	13.6	−12.0	non-VGIIa	23.9	14.9	−9.0	non-VGIIb	18.0	31.5	13.4	VGIIc	**VGIIc**
B9235	VGIIc	25.9	13.7	−12.1	non-VGIIa	24.1	14.9	−9.2	non-VGIIb	18.4	32.4	14.0	VGIIc	**VGIIc**
B9244	VGIIc	27.2	19.1	−8.1	non-VGIIa	26.2	16.9	−9.2	non-VGIIb	20.2	32.5	12.3	VGIIc	**VGIIc**
B9245	VGIIc	28.4	22.9	−5.5	non-VGIIa	25.2	17.4	−7.8	non-VGIIb	20.7	34.5	13.8	VGIIc	**VGIIc**
B9295	VGIIc	21.0	17.1	−3.8	non-VGIIa	26.0	19.6	−6.4	non-VGIIb	22.1	28.1	5.9	VGIIc	**VGIIc**
B9302	VGIIc	26.7	15.6	−11.1	non-VGIIa	23.7	15.4	−8.3	non-VGIIb	19.4	34.3	15.0	VGIIc	**VGIIc**
B9374	VGIIc	27.4	21.6	−5.8	non-VGIIa	24.0	15.3	−8.7	non-VGIIb	19.4	33.4	14.0	VGIIc	**VGIIc**

**Table 6:** Interassay and Intraassay for MLST and Subtyping MAMA

**Assay**	**interrun CV (%)**	**intrarun CV (%)**
VGI	4.33	1.56
VGII	2.35	0.22
VGIII	0.43	0.60
VGIV	1.37	1.08
VGIIa	0.22	0.50
VGIIb	1.27	0.92
VGIIc	1.61	0.32

**Table 7:** Lower limit dynamic range for MLST and subtyping MAMA primer sets

**Primer set tested**	**Limit (pg)**	**Median Ct**
VGI	0.5	31.7
non-VGI	0.5	31.1
VGII	0.5	29.5
non-VGII	0.5	28.7
VGIII	0.5	28.5
non-VGIII	0.5	29.9
VGIV	0.5	33.7
non-VGIV	0.5	33.2
VGIIa	0.5	30.2
non-VGIIa	0.5	31.2
VGIIb	0.5	30.1
non-VGIIb	0.5	28.5
VGIIc	0.5	37.4
non-VGIIc	0.05	39.4

## Discussion

*C. gattii* is an emerging pathogen in the US Pacific Northwest and British Columbia. Molecular and epidemiological investigations revealed the Vancouver Island, BC outbreak was attributed to a novel and seemingly hypervirulent VGIIa genotype [7,20,22]; moreover, the recent PNW outbreak was attributed to an additional novel genotype, VGIIc [23]. These apparent new genotypes (VGIIa and VGIIc), are responsible for greater than 90% of *C. gattii* infections in the BC/PNW region [7]. Given the increased virulence, varying antifungal susceptibilities and clinical outcomes caused by these genotypes, as compared to other *C. gattii* genotypes, it will be useful to conduct regular genotyping of *C. gattii* isolates for both clinical and epidemiological response purposes [5,7,9,16].

We have developed a MAMA real-time PCR panel for cost-efficient and rapid genotyping of *C. gattii* molecular types (I-IV) and VGII subtypes (a-c) as a means to better understand genotype distribution of *C. gattii* in North America. To validate the assays, we screened DNA from a diverse North American and international isolate collection of *C. gattii* isolates from human, environmental, and animal sources. All DNA had been previously typed by MLST. The assay panel performed with 100% sensitivity and specificity and was 100% concordant with MLST results. The VGII subtype specific assays may be more pertinent to the North American public health and medical communities; the molecular type (I-IV) specific assays will be useful for both North American and global genotyping. The assay is designed for screening in a cost-effective, step-wise manner. The molecular type-specific assays should be performed first on all isolates. In North America, the VGIV assay can be withheld for the first screen, as isolates of this molecular type have not yet been isolated from North America. For those North American isolates that are VGII by molecular type, the subtype-specific assays should be performed for typing VGIIa, VGIIb, or VGIIc. As we further our understanding of *C. gattii* populations around the world and their genotype-phenotype relationships, additional subtype specific assays can be similarly developed for local and global research purposes.

## Conclusions

These PCR-based assays are an affordable, efficient, and sensitive means of genotyping *C. gattii* isolates. Both the assay methods and results can be easily transferred among laboratories. Assay results are based on real-time PCR cycle threshold values and are therefore objective and straightforward for local analysis. The assay panel presented here is a useful tool for conducting large-scale molecular epidemiological studies by public health and research laboratories.

### Ethics statement

This study does not involve subjects or materials that would require approval by an ethics committee.

## Abbreviations

MAMA: Mismatch amplification mutation assay; MLST: Multilocus sequence typing; PCR-RFLP: PCR-restriction fragment length polymorphism; AFLP: Amplified fragment length polymorphism; MLMT: Multilocus microsatellite typing; HRM: High resolution melting; MALDI-TOF MS: Matrix-assisted laser desorption ionization-time-of-flight mass spectrometry; ASPCR: Allele-specific PCR; SNP: Single nucleotide polymorphism; Ct: Cycle threshold; MPD1: *Mannitol-1-phosphate dehydrogenase*; WGST: Whole genome sequence typing.

## Competing interests

The authors declare that they have no competing interests.

## Authors’ contributions

EK designed the assays, assisted with assay validation, data analysis and drafted the manuscript. EMD participated in the design and coordination of the study, data analysis and assisted with drafting the manuscript. KE performed assay validation and data analysis and assisted with drafting the manuscript. MB was involved in the study conception, design and coordination. JS and JG assisted with data analysis for study design. JT performed assay validation and assay data analysis. SL and ED assisted with study conception, design and coordination and manuscript review. PK assisted with study design, coordination and manuscript review. DE assisted with study conception, design, coordination, and drafting of the manuscript. All authors read and approved the final manuscript.
